# The effect of experimental warming on leaf functional traits, leaf structure and leaf biochemistry in *Arabidopsis thaliana*

**DOI:** 10.1186/1471-2229-11-35

**Published:** 2011-02-18

**Authors:** Biao Jin, Li Wang, Jing Wang, Ke-Zhen Jiang, Yang Wang, Xiao-Xue Jiang, Cheng-Yang Ni, Yu-Long Wang, Nian-Jun Teng

**Affiliations:** 1 College of Biological Sciences and Biotechnology, Yangzhou University, Yangzhou 225009, PR China; 2 College of Horticulture, Nanjing Agricultural University, Nanjing 210095, PR China; 3 Key Laboratory of Photosynthesis and Environmental Molecular Physiology, Institute of Botany, Chinese Academy of Sciences, Beijing 100093, PR China; 4 College of Horticulture and Plant Protection, Yangzhou University, Yangzhou 225009, PR China; 5 Key Laboratory of Crop Genetics and Physiology of Jiangsu Province, Yangzhou University, Yangzhou 225009, PR China

## Abstract

**Background:**

The leaf is an important plant organ, and how it will respond to future global warming is a question that remains unanswered. The effects of experimental warming on leaf photosynthesis and respiration acclimation has been well studied so far, but relatively little information exists on the structural and biochemical responses to warming. However, such information is very important to better understand the plant responses to global warming. Therefore, we grew *Arabidopsis thaliana *at the three day/night temperatures of 23/18°C (ambient temperature), 25.5/20.5°C (elevated by 2.5°C) and 28/23°C (elevated by 5°C) to simulate the middle and the upper projected warming expected within the 21st century for this purpose.

**Results:**

The 28/23°C treatment significantly reduced the life span, total biomass and total weight of seeds compared with the other two temperatures. Among the three temperature regimes, the concentrations of starch, chlorophyll, and proline were the lowest at 28/23°C, whereas the total weight of seeds, concentrations of chlorophyll and proline, stomatal density (SD), stomatal conductance (g_s_), net CO_2 _assimilation rate (A) and transpiration rate (E) were the highest at 25.5/20.5°C. Furthermore, the number of chloroplasts per cell and mitochondrial size were highest at 25.5/20.5°C and lowest at 28/23°C.

**Conclusions:**

The conditions whereby the temperature was increased by 2.5°C were advantageous for *Arabidopsis*. However, a rise of 5°C produced negative effects, suggesting that lower levels of warming may benefit plants, especially those which belong to the same functional group as *Arabidopsis*, whereas higher levels of warming may produce negative affects. In addition, the increase in A under moderately warm conditions may be attributed to the increase in SD, chlorophyll content, and number of chloroplasts. Furthermore, starch accumulation in chloroplasts may be the main factor influencing chloroplast ultrastructure, and elevated temperature regulates plant respiration by probably affecting mitochondrial size. Finally, high SOD and CAT activities may enable plants grown at elevated temperatures to exhibit relatively high tolerance to temperature stress, thus alleviating the harmful effects of superoxide anion radicals and hydrogen peroxide.

## Background

Atmospheric concentrations of greenhouse gases such as CO_2_, CH_4_, and N_2_O have increased dramatically since the beginning of the industrial revolution due to fossil fuel combustion, deforestation and land development; together, these probably led to a rise in ground-level air temperatures at an unprecedented rate over the past three decades [1,2]. Moreover, the global mean temperature will continue to rise at a rapid rate, and our climate is likely to warm by 1.1-6.4°C within the next century [2]. Most plant species only grow in a certain temperature range. Thus, some are likely to adapt to warmer temperatures by changing their growth and development or by shifting their ranges, provided that the optimum temperatures are not exceeded. Some species may fail to adapt to this global change and may even become extinct if the air temperature is too high [3-5]. Therefore, projected atmospheric warming is expected to have profound effects on plant physiology and growth, structure and function of plant populations, species distributions, and probabilities of extinction [6,7]. Moreover, this change in plants may result in complex impacts on vegetation and biodiversity, leading to terrestrial ecosystem consequences [8,9]. Thus, understanding the changes in plant growth and development in response to simulated climatic warming is important to predict plant responses to global warming in the near future.

Many studies have investigated plant responses to global warming at different scales, with most performed at community level, and only a few at the individual level or a focus on responses of leaves to temperature increase [5,10]. Because the leaf is the key organ performing photosynthesis and transpiration, its development, which varies with environmental factors, is an important determinant of total plant productivity [11]. In addition, leaves can be indicators of plant community responses to global warming, because their responses are not only the basis of changes at the community level, but they are among those organs that show visible impacts of air temperatures [1,12]. Furthermore, leaf traits can express phenotypically plastic responses to growth temperature [13]. Consequently, experiments on the effects of global warming on leaf growth and development will provide a better understanding of the mechanism of plant responses to global warming at the community level.

Previous studies mainly investigated the effects of experimental warming on leaf photosynthesis and respiration acclimation, but leaf structure (microstructure and ultrastructure) and biochemical processes were seldom focused on [1,11,14]. Because leaf structure is one of the most important traits exhibiting phenotypic plasticity to growth temperature, investigating responses of leaf structure to warming is fundamental to projecting the impact of global change on plant growth. In addition, leaf biochemical and physiological changes are related to leaf structure and function. For example, temperature stress is known to induce plants to produce reactive oxygen species (ROS) and malondialdehyde (MDA), which can damage both the leaf structure and function [15,16]. To alleviate the damage, plants generally enhance the production of ROS scavenging enzymes, such as superoxide dismutase (SOD) and catalase (CAT), and osmoprotectants like proline and carbohydrates. Although many studies have investigated the effects of high temperature on the production of antioxidant enzymes and osmoprotectants, the periods of high temperature were usually limited to several hours or days; also, few studies examined these biochemical and physiological changes under global warming conditions for one generation [17-19]. Therefore, to obtain an integrative understanding of the responses of leaf growth to global warming, we examined the effects of simulated climatic warming on SOD and CAT activities, contents of MDA, proline, carbohydrates and chlorophyll of *Arabidopsis thaliana *leaves, and leaf microstructure and ultrastructure, apart from fitness components. *Arabidopsis *is a model plant widely used in molecular, genetic, and developmental biology. Therefore, studying its responses may represent a valuable assessment of the possible plant changes occurring at the individual level in a future warmer world.

## Methods

### Experimental design and growth conditions

Seeds of *A. thaliana *(L.) Heynh. [Wild-type Columbia (Col-0), Nottingham *Arabidopsis *Stock Centre, Nottingham University, UK] were exposed to stratification at 4°C for 2 d before planting. Then they were sown in 400-cm^3 ^plastic pots containing a 1:1 (v/v) mixture of vermiculite and peat (Kaiyin Company, Beijing, China). The plants were grown in growth chambers (RXZ-300B, Ningbo Dongnan Instruments Co Ltd, China). The middle and upper projected warming in the 21st century is expected to approximate 2.5 and 5°C, respectively [2]. This ecotype originally derives from Columbia in USA, and the spring/autumn average temperature in this location is 15-16/21-22°C http://www.arabidopsis.org/servlets/TairObject?type=species_variant&id=90. The common growth temperature for this ecotype is 22-23°C/16-19°C (day/night) in many laboratories, and this nearly corresponds to growth temperatures in nature. In addition, some studies have used 23°C as the baseline or ambient temperature to investigate the effects of temperature on *Arabidopsis *flowering [20,21]. Furthermore, the seeds used here were obtained from plants that have grown in growth chambers at 23/18°C for more than ten generations by seed propagation over the past several years. Consequently, this ecotype may have adapted to this growth temperature after so many generations were grown at 23/18°C. Therefore, in the present study, the day/night temperatures in the growth chambers were maintained at 23/18°C, and this is referred to as 'ambient temperature', whereas 25.5/20.5°C is 'elevated temperature I', and 28/23°C is 'elevated temperature II', respectively ((with 1 growth chamber per temperature regime). The results from such experiments will help to predict the responses of plants to the future middle and upper warming regimes. The plants were grown under a 16-h photoperiod and 500 μmolm^-2 ^s^-1 ^of photosynthetically active radiation (PAR), provided by fluorescent tubes (Philips Electronics Trading & Services Co Ltd, Shanghai, China), at 80/95% RH (day/night). Every week, the plants were alternately watered to saturation with 1/2 MS solution or de-ionized water. The seedlings were thinned to one individual closest to the center of each pot after emergence. The pots were randomly rearranged every 3 d to negate any possible effects of position within the chambers. When bolting had just commenced (i.e. stage 5.10) [22], the leaves were sampled for the following analyses, with all analyses repeated on five plants. When over 95% of the siliques were mature (i.e. stage 9.70) [22], all the plant material was sampled. Except for the seeds, all other plant material was dried to a constant weight at 60°C and then measured on an electronic balance. The seeds were weighed after they were stored in a desiccator at room temperature for over 20 days. The life span and total biomass were then calculated based on 35 plants per treatment.

### Gas exchange measurements and determination of stomatal density

Three fully expanded leaves from each of five plants per treatment were selected during the middle of the light period to measure the stomatal conductance (g_s_), transpiration rate (E), and net CO_2 _assimilation rate (A) using an LI-6400 Portable Photosynthesis System (LI-COR Inc., Lincoln, Nebraska, USA). The measurements were carried out at 1500 μ mol m^-2 ^s^-1 ^PAR, 2.0-2.5 KPa VPD, 23°C, and 370-390 ppm CO_2_. The stomatal density (SD) was determined as outlined by Ceulemans et al. [23]; three leaves per plant were sampled from five plants, and 20 separate fields of 0.16 mm^2 ^were analyzed per leaf [24].

### Determination of carbohydrate, protein, and chlorophyll contents

Soluble sugars were extracted from leaf tissue by hot ethanol extraction, and starch was extracted from the pellet as follows. Leaves were sampled at the end of the light period, oven-dried at 60°C, and homogenized. Approximately 50 mg of dry leaf power of each sample was extracted with 80% ethanol (v/v) at 85°C for 60 min. The extracts were then centrifuged at 12,000 g for 10 min. The ethanol extraction step was repeated three times. The three resulting supernatants were combined, treated with activated charcoal, and evaporated to dryness in a vacuum evaporator. The residues were redissolved in distilled water and subjected to soluble sugar analysis using the anthrone-sulfuric acid method [25]. Following the removal of soluble sugars, the remaining residues were oven-dried overnight at 60°C and then subjected to starch analysis according to the procedures described in Vu et al. [26].

Leaf protein concentrations were determined according to Bradford [27] using bovine serum albumin as the standard. Chlorophyll *a *and *b *were extracted with the acetone method. After 0.5 g of leaf tissue was homogenized in 5 mL of 100% acetone, the extract was added to 5 mL of 80% (v/v) acetone and then centrifuged at 12,000 g for 10 min. The absorbance of the supernatant was read at 663 nm and 645 nm, respectively. The chlorophyll *a *and b contents were calculated according to the method of Porra [28].

### Measurements of MDA, proline, and enzyme activity

MDA in leaves was measured by the thiobarbituric acid (TBA) method [29] with slight modifications. Fresh leaves (~ 0.5 g) were homogenized with a mortar and pestle in 10% (w/v) trichloroacetic acid. Then the homogenate was centrifuged at 12,000 g for 10 min. Two mL of supernatant were mixed with 2 mL of 10% trichloroacetic acid containing 0.5% (w/v) thiobarbituric acid. The mixture was boiled at 100°C for 30 min and then quickly cooled in an ice bath. After centrifugation at 12,000 g for 10 min at 4°C, the supernatant absorbance was read at 532 nm, and values corresponding to non-specific absorption at 600 nm were subtracted. The MDA concentration was calculated using its extinction coefficient (155 mM^-1 ^cm^-1^).

The extraction and content determination of proline in leaves was performed according to the method of Bates et al [30]. Fresh leaves (~ 0.5 g) were homogenized in 10 mL of 3% aqueous sulfosalicylic acid, and the extracts were centrifuged at 4000 g for 10 min. Two mL of supernatant were reacted with 2 mL of 2.5% acidic ninhydrin and 2 mL glacial acetic acid in a test tube for 1 h at 100°C; the reaction was terminated in an ice bath. The reaction mixture was extracted with 4 mL of toluene, mixed thoroughly, and warmed to room temperature. The absorbance was read at 520 nm using toluene as a blank, and the proline concentration was calculated.

The methods for determining the SOD and CAT activities are listed next. The total rosette leaves were sampled and immediately frozen in liquid nitrogen after fresh weight was measured, and then stored at -80°C until further use. A 0.5-g sample of leaf tissue was homogenized in 10 mL of 0.1 mol/L phosphate buffer (pH 7.8) supplemented with 1% (w/v) polyvinylpyrrolidone and then centrifuged at 12,000 g for 15 min. The supernatants were used for enzyme assays. All steps of the extraction procedure were carried out at 0-4°C. The SOD activity was measured according to the method of Beauchamp and Fridovich [31] with minor modifications. The reaction mixture (3 mL) contained 13 mmol/L methionine, 75 μmol/L nitroblue tetrazolium (NBT), 2.0 μmol/L riboflavin, 0.1 mmol/L EDTA, and 0.1 mL of enzyme extract in 50 mmol/L phosphate buffer (pH 7.8). Glass test tubes containing the reaction mixture were illuminated with a fluorescent lamp for 15 min at 25°C. Non-illuminated and illuminated reactions without the enzyme extract served as calibration standards. After illumination, the photoreduction of NBT (production of blue formazan) was measured at 560 nm using a Beckman spectrophotometer (DU 640, Beckman Coulter, Germany). One unit of SOD was defined as the enzyme activity that inhibited the photoreduction of NBT to blue formazan by 50%. The CAT activity was determined at 25°C by following the method of Claiborne [32] with slight modifications. The reaction mixture (3 mL) contained 10 mmol/L H_2_O_2 _and 0.2 mL of enzyme extract in 50 mmol/L phosphate buffer (pH 7.0). The CAT activity was determined based on the decrease in absorbance of H_2_O_2 _at 240 nm.

### Leaf structural observation

At every temperature, three fully expanded leaves from each of five plants were dissected and immediately fixed in 2.5% (v/v) glutaraldehyde (in 0.1 mol/L phosphate buffer, pH 7.0) for 2 h at 4°C. Then the samples were washed five times with the same buffer and post-fixed in 1% osmium tetroxide for 3 h. After being washed with the same buffer, the leaf tissues were passed through an ethanol dehydration series, infiltrated, and embedded in Spurr's resin. The embedded leaf tissues were sectioned with an LKB-V ultramicrotome (Bromma, Sweden). The 1-μm-thick sections were stained with 1% toluidine blue O in 2% sodium borate for general tissue staining; they were then observed and photographed under a microscope (Zeiss Axioskop 40: Carl Zeiss Shanghai Company Limited, Shanghai, China). At each temperature, three leaves from each of five plants were sampled for measuring the leaf thickness and number of cell layers. The cell size was calculated using AutoCAD 2004 (Autodesk, Inc, USA) from digital pictures. In addition, sections were cut using an LKB-V ultramicrotome. Thin sections were stained with uranyl acetate and lead citrate; they were then observed and photographed under a transmission electron microscope (JEOL Ltd, Tokyo, Japan) [24]. For each treatment, the cell (the cells in palisade and spongy tissues) size and number of chloroplasts per cell were determined from 300 cells. Chloroplast length and width, area of chloroplast profile, and ratio of total starch grains per chloroplast relative to chloroplast area were determined from 100 chloroplasts. The area per starch grain was determined from 100 starch grains, and the mitochondrial length and width were determined from 100 mitochondria.

### Statistical analysis

The data are shown as the mean values ± standard deviation. The data were subjected to a one-way analysis of variance using the SPSS software 16.0 (SPSS Inc, Chicago, IL, USA), and the means were compared using the Bonferroni t-test with alpha = 0.05 (the type I experimentwise error rate).

## Results

### Life span and plant biomass

**Figure 1:**
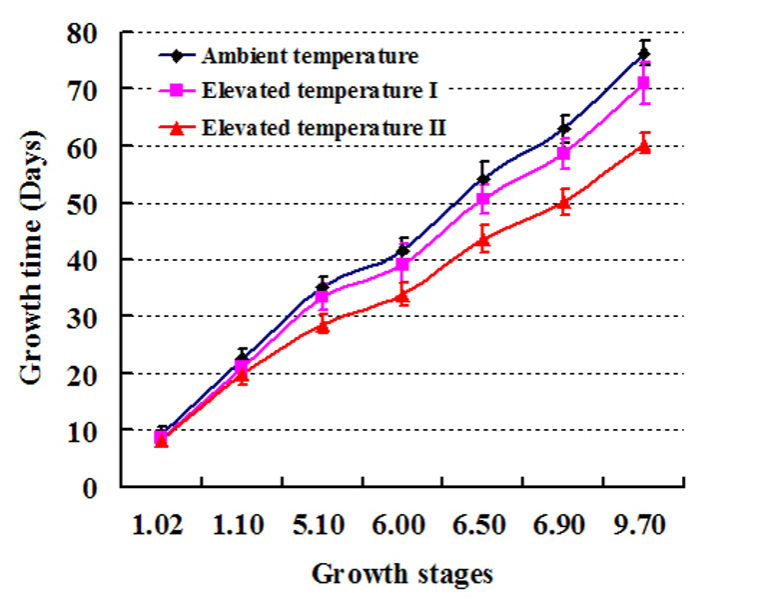
**Growth curves of *Arabidopsis *grown at three temperatures**. The growth stages 1.02, 1.1, 5.1, 6.00, 6.50, 6.90, and 9.70 correspond to "2 rosette leaves >1 mm in length", "10 rosette leaves >1 mm in length", "first flower buds visible", "first flower open", "50% of flowers to be produced have opened", "flowering complete", and "senescence complete", respectively (Please refer to Table two (p. 1501) and Figure two (p. 1502) of Boyes *et al*. 2001 [22]).

Experimental warming markedly enhanced *Arabidopsis *growth and shortened its life span (Figure 1, Table 1). For example, when compared with ambient temperature, elevated temperatures I and II significantly shortened the life span of *Arabidopsis *by approximately 7% and 21%, respectively. There was no significant difference in the plant biomass between ambient temperature and elevated temperature I, but elevated temperature II significantly reduced it by about 35% compared with the other two temperatures. Relative to ambient temperature, elevated temperature I significantly increased total weight of seeds by approximately 37%, whereas elevated temperature II reduced it by approximately 14%.

**Table 1:** Effects of experimental warming on *Arabidopsis*

Growth, physiological, biochemical and structural parameters	Ambient temperature (23/18°C)	Elevated temperature I (25.5/20.5°C)	Elevated temperature II (28/23°C)
Life span (days)	76.2 ± 2.2a	71.0 ± 3.6b	60.4 ± 1.9c
Plant biomass (mg)	2128.0 ± 349.4a	2116.7 ± 337.7a	1378.5 ± 370.4b
Total weight of seeds (mg)	392.5 ± 110.7b	536.6 ± 139.6a	338.5 ± 114.9b
SD (number per mm^2^) on the adaxial surface	181 ± 13b	224 ± 15a	200 ± 14ab
SD (number per mm^2^) on the abaxial surface	206 ± 14b	265 ± 16a	214 ± 13b
g_s _(m mol m^-2 ^s^-1^)	378 ± 24b	423 ± 28a	385 ± 21ab
E (m mol m^-2 ^s^-1^)	7.5 ± 0.4b	8.4 ± 0.6a	7.7 ± 0.4b
A (μ mol m^-2 ^s^-1^)	14.3 ± 1.1b	16.5 ± 1.4a	12.4 ± 0.9c
Soluble sugars (μgmg^-1 ^DW)	38.7 ± 3.0ab	35.2 ± 1.3b	40.0 ± 2.0a
Starch (μgmg^-1 ^DW)	61.4 ± 5.7a	53.7 ± 2.3b	48.8 ± 4.3b
Protein (μgmg^-1 ^DW)	19.7 ± 1.9a	20.0 ± 2.3a	22.0 ± 2.2a
Chlorophyll a (mg g^-1 ^FW)	0.93 ± 0.05b	1.04 ± 0.07a	0.84 ± 0.04b
Chlorophyll b (mg g^-1 ^FW)	0.30 ± 0.02ab	0.34 ± 0.03a	0.26 ± 0.03b
Chlorophyll a+b (mg g^-1 ^FW)	1.23 ± 0.06b	1.38 ± 0.09a	1.10 ± 0.07b
Chlorophyll a/b	3.1 ± 0.2a	3.1 ± 0.2a	3.2 ± 0.2a
MDA (nmol g^-1 ^FW)	2.3 ± 0.2b	2.0 ± 0.2b	3.8 ± 0.5a
Proline (ug g^-1 ^FW)	15.3 ± 2.1b	22.8 ± 3.2a	14.4 ± 1.7b
SOD (Unit g^-1 ^FW)	295 ± 16b	347 ± 25a	319 ± 18ab
CAT (Unit g^-1 ^FW min^-1^)	7.9 ± 1.1b	16.1 ± 1.5a	17.7 ± 1.9a
Leaf thickness (μm)	159 ± 14a	155 ± 14ab	146 ± 13b
Cell Size (μm^2^)	981 ± 398a	939 ± 372a	774 ± 337b
Number of cell layer	7.6 ± 1.1a	7.5 ± 1.2a	7.3 ± 1.2a
Number of chloroplasts per cell	8.5 ± 2.2a	9.0 ± 2.8a	6.6 ± 2.5b
Chloroplast length (μm)*	5.0 ± 1.3a	5.1 ± 1.4a	5.1 ± 1.3a
Chloroplast width (μm)*	2.3 ± 0.5a	1.9 ± 0.4b	1.6 ± 0.4c
Area of chloroplast profile (μm^2^)	9.2 ± 5.0a	7.8 ± 4.0b	6.7 ± 3.5b
Area per starch grain (μm^2^)	1.2 ± 0.7a	0.5 ± 0.3b	0.5 ± 0.3b
Ratio of total starch grains per chloroplast relative to chloroplast area (%)	29 ± 9a	15 ± 5b	13 ± 5b
Mitochondrial length (μm)*	0.7 ± 0.2b	0.9 ± 0.2a	0.7 ± 0.1b
Mitochondrial width (μm)*	0.5 ± 0.1b	0.6 ± 0.1a	0.5 ± 0.1b

Values (mean ± standard deviation) with the same letter are not significantly different at α = 0.05 by the Bonferroni t-test. *The length of chloroplasts and mitochondria is the longest dimension, and the width of chloroplasts and mitochondria is the widest dimension. SD: stomatal density; g_s_: stomatal conductance; E: transpiration rate; A: net CO_2 _assimilation rate; DW: dry weight; FW: fresh weight.

### Stomatal and photosynthetic characters

Compared with ambient temperature, the SD on the adaxial and abaxial surfaces at elevated temperature I was significantly increased by 24% and 29%, respectively. However, no significant difference in SD was observed between ambient temperature and elevated temperature II (Table 1). In addition, elevated temperature I also significantly enhanced g_s_, E, and A relative to ambient temperature. For instance, g_s_, E, and A at elevated temperature I were increased by 12%, 12%, and 15%, respectively (Table 1). There was no significant difference in g_s _and E between ambient temperature and elevated temperature II, but A was significantly reduced by about 13% at elevated temperature II compared to ambient temperature.

### Levels of carbohydrates, protein, and chlorophyll

Temperatures profoundly affected the leaf soluble sugar and starch contents. Compared with ambient temperature, the foliar content of soluble sugars at elevated temperature I was reduced by approximately 9%, but there was no significant difference in the content of soluble sugars between ambient temperature and elevated temperature I. The foliar content of soluble sugars did not differ significantly between ambient temperature and elevated temperature II. Compared to elevated temperature I, the content of soluble sugars at elevated temperature II was increased by 13%. The starch content of leaves was highest at ambient temperature and was followed by elevated temperatures I and then II. There was no significant difference in the protein content among the three temperatures. Relative to ambient temperature, elevated temperature I increased the contents of chlorophyll *a *and *b*, whereas lower values were recorded at elevated temperature II. The ratio of chlorophyll *a *to *b *at all three temperatures was approximately 3:1 and was not markedly affected by temperature (Table 1).

### MDA and proline contents and enzyme activity

Temperature influenced the MDA and proline contents in leaves. The foliar MDA content was significantly higher at elevated temperature II than at the other temperatures. Compared with ambient temperature, elevated temperature I slightly decreased the foliar MDA content by 13%, whereas elevated temperature II significantly increased its content by approximately 65%. The proline content at elevated temperature I was higher than that at ambient temperature and elevated temperature II by 63% and 67%, respectively. However, there was no significant difference in the proline content between ambient temperature and elevated temperature II (Table 1).

Relative to ambient temperature, elevated temperature I significantly increased the SOD activity by 18%, whereas elevated temperature II slightly increased the SOD activity by 8%. However, there was no significant difference in SOD activity between elevated temperatures I and II. There was a positive correlation between CAT activity and temperature. In comparison with ambient temperature, the CAT activity at elevated temperatures I and II was significantly increased by 104% and 124%, respectively. However, there was no significant difference in the CAT activity between elevated temperatures I and II, although the CAT activity for the latter was 10% higher than the former (Table 1).

### Leaf microstructure and ultrastructure

**Figure 2:**
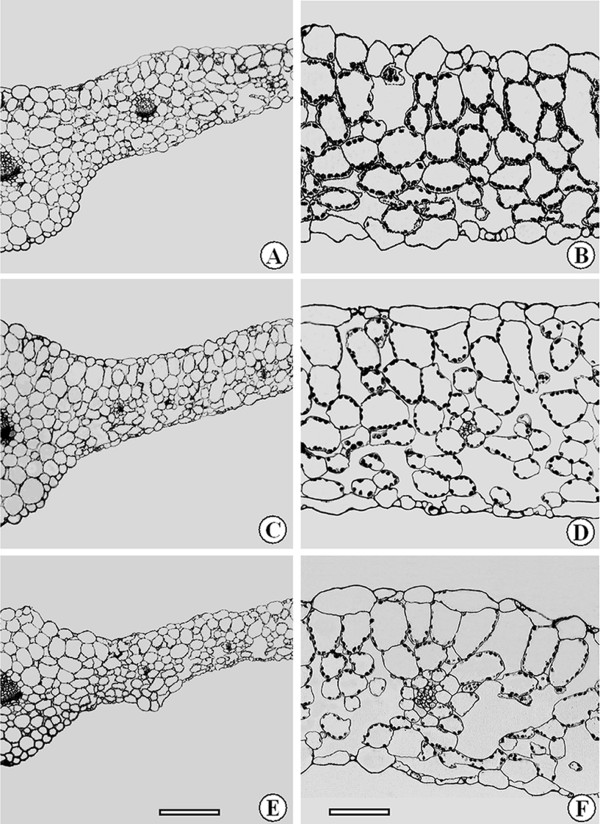
**Cross sections of leaves of *Arabidopsis *grown at three temperatures**. Samples were taken at ambient temperature (A and B), elevated temperature I (C and D), and elevated temperature II (E and F). Note that the leaf at elevated temperature II was the thinnest of the three temperatures. In addition, there were more chloroplasts per cell at ambient temperature and elevated temperature I than elevated temperature II. Bars, 150 μm (A, C and E); 50 μm (B, D and F).

Leaf thickness and cell size were not significantly different between ambient temperature and elevated temperature I, but at elevated temperature II they were significantly reduced by approximately 8.2% and 21.1%, respectively, compared to those at ambient temperature. However, no difference was observed in the number of cell layers among the three temperatures. Therefore, the changes in leaf thickness were mainly due to changes in cell size since the number of cell layers was not markedly affected by temperature (Table 1, Figure 2).

Relative to ambient temperature, elevated temperature II caused a decrease of 22% in the number of chloroplasts per mesophyll cell, but there was no significant difference between ambient temperature and elevated temperature I. In addition, chloroplast length was not significantly influenced by temperature, but chloroplast width was. For instance, compared with ambient temperature, chloroplast width at elevated temperatures I and II was decreased by 17% and 30%, respectively (Table 1, Figure 2A-C). Chloroplast width at elevated temperature I was 16% higher than at elevated temperature II. Given the unchanged chloroplast length, the concomitant reduction in chloroplast profile area was a result of the decreased widths at elevated temperatures I and II.

**Figure 3:**
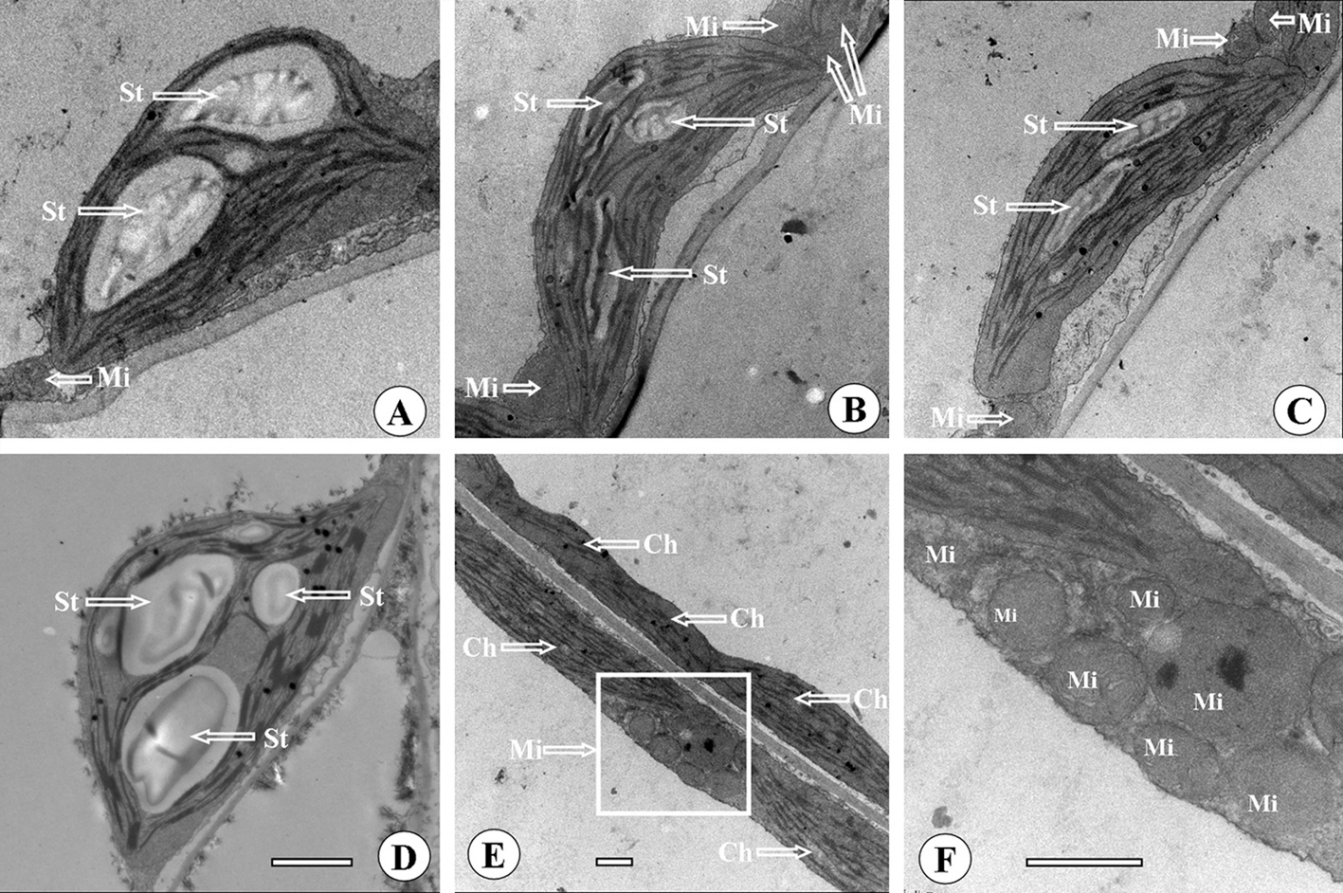
**Transmission electron micrographs showing leaf chloroplast and mitochondrial ultrastructure of *Arabidopsis *grown at three temperatures**. Samples were taken at ambient temperature (A and D), elevated temperature I (B, E and F), and elevated temperature II (C). Note that there were larger starch grains in the chloroplasts of *A. thaliana *leaves grown at ambient temperature than at elevated temperatures I and II. In addition, there were more mitochondria nearby chloroplasts at elevated temperatures I and II than at ambient temperature. St, starch grain; Mi, mitochondrion; Ch, chloroplast. Bar, 1 μm (A-F).

The size of starch grains and the ratio of total starch grains per chloroplast relative to the chloroplast profile area at ambient temperature were dramatically higher than those at elevated temperatures I and II. The average size per starch grain decreased from 1.2 μm^2 ^at ambient temperature to approximately 0.5 μm^2 ^at both elevated temperatures I and II (Table 1, Figure 3A-D). Starch grains accounted for an average of 15% and 13% of the chloroplast profile at elevated temperatures I and II, respectively; these values were lower than the 29% at ambient temperature (Table 1, Figure 3A-C). At ambient temperature, the starch grains took up approximately 50% of the chloroplast profile (Figure 3D). About 40% of chloroplasts lacked starch grains at elevated temperatures I and II compared to approximately 25% at ambient temperature.

The size and number of mitochondria were affected by temperature. Mitochondria were larger at elevated temperature I than at the other two temperatures (Table 1, Figure 3A-C). For example, relative to ambient temperature, elevated temperature I significantly increased mitochondrial length and width by 29% and 20%, respectively. However, there was no difference in mitochondrial size between ambient temperature and elevated temperature II. In general, there were more mitochondria near chloroplasts at elevated temperatures I and II than at ambient temperature (Figure 3A-D). It was interesting that chloroplasts contained few starch grains at elevated temperatures I and II when many mitochondria were near chloroplasts (Figure 3E, F). Thus, there was a negative relationship between the size and number of starch grains in chloroplasts and the number of mitochondria near the chloroplasts.

## Discussion

### Plant growth and optimum growth temperature

The growth temperature range for *Arabidopsis *is 21-23°C in most laboratories, but this is higher than its minimum growth temperature. Compared with vegetative growth, the *Arabidopsis *reproductive growth (especially after fertilization of most flowers) can tolerate higher temperatures, because older plants are usually less sensitive to temperature than younger ones [33]. Our results show that 23°C is below the optimum temperature for the growth of *Arabidopsis*, because the plants grew better at 25.5°C than at 23°C. However, a temperature of 28°C negatively affected leaf growth and significantly reduced the total biomass and total weight of seeds. Therefore, 25.5°C is closer to the optimum *Arabidopsis *growth temperature, and 28°C is clearly above the optimum level for growth. The results of this warming experiment using *Arabidopsis*, a small annual herb with short life cycle, may be useful for predicting how plants, especially those belonging to the same functional group as *Arabidopsis*, respond to an increasing air temperature. For example, some annual herbs might benefit from low levels of warming that do not exceed their optimum growth temperature; in contrast, higher levels of warming may produce negative effects since plants that belong to the same functional group usually respond in similar ways to changes in environmental factors [34,35].

### Photosynthetic and stomatal characteristics

A large body of work has shown that climatic warming can stimulate plant photosynthesis and increase plant productivity [36,37]. Compared to the measurements at ambient temperature, the chlorophyll content and A at elevated temperature I increased by 12% and 15%, respectively, consistent with previous reports. Increased A may be due to the increased chlorophyll content and g_s_, because the chlorophyll content and g_s _are usually positively correlated to A [38]. However, relative to ambient temperature, elevated temperature II had a significantly lower A and chlorophyll content, but g_s _was not significantly affected; this result is in contrast with some findings reporting that experimental warming increased A [37,39]. This apparent discrepancy may be partly attributable to differences in the extent of temperature increase, i.e. a rise of 0-3°C in the previous studies compared to 5°C at elevated temperature II. The temperature used in the previous experiments may not have exceeded the optimum temperature of photosynthesis, whereas elevated temperature II may have. When the temperature exceeds optimum range, A declines by reducing the activation of ribulose-1,5-bis-phosphate carboxylase/oxygenase [40]. In addition, the significant reduction in the number of chloroplasts per cell at elevated temperature II may be also a reason causing lower A. In the present study, the significant decrease in plant biomass at elevated temperature II may be a direct effect of decreased A and a shorter life span. Although A was significantly higher at elevated temperature I compared to ambient temperature, there was no significant difference in plant biomass between them. The first reason accounting for this could be the shorter life span of the plants at elevated temperature I compared to ambient temperature, as well as the advantage of higher A at elevated temperature I being offset by a shorter growth time. Secondly, plants grown at elevated temperature I may have had a higher E in the darkness, thus consuming higher amounts of soluble sugars and starch compared with those grown at ambient temperature.

### Activities of antioxidant enzymes and MDA content

Temperature stress is known to induce plants to produce reactive oxygen species (ROS) and MDA, both of which can damage tissues [15,16]. To ensure survival, plants generally enhance the production of ROS scavenging enzymes, such as SOD and CAT, and osmoprotectants like proline [16,17]. In the present study, the MDA content recorded at elevated temperature II was the highest of the three temperatures, indicating that high temperature stress negatively affected the plants. However, no significant differences were observed in the SOD and CAT activities between elevated temperature I and II. This result could be attributed to the following reasons. The high SOD and CAT activities enabled the plants grown at elevated temperature I to exhibit a relatively high tolerance to temperature stress, possibly accounting for their fast growth. For the plants grown at elevated temperature II, the high enzyme activities may enable them to quickly clear superoxide anion radicals and catalyze the decomposition of hydrogen peroxide to water and oxygen, thus alleviating the harmful effects of these detrimental products. Therefore, high SOD and CAT activities at elevated temperature II may be a positive feedback or protection mechanism that is triggered when the plant is subjected to relatively severe long-term warming stress. The proline content, an indicator of resistance to heat stress, was the lowest at elevated temperature II. It is possible that less proline was produced because of the partially inhibition of normal metabolic capability at elevated temperature II. However, plants at elevated temperature I may have a less-affected heat-resistant system that produces more proline as a tolerance mechanism to heat stress, given that the proline content was the highest at this temperature.

### Leaf structure

Among the three temperatures, the number of chloroplasts was greatest at elevated temperature I and lowest at II. The number of chloroplasts was proportional to the chlorophyll content and A, indicating a concomitant change in chloroplast number, chlorophyll content, and photosynthesis. Our results are in agreement with the general notion of a close correlation between A and chloroplast number [41]. Similar findings have been reported for the effects of elevated CO_2 _on chloroplast number [42]. Chloroplast width was mainly influenced by starch accumulation, and the chloroplast profile area was largely affected by its width, since its length did not vary much. In fact, increased starch accumulation widened leaf chloroplasts in previous reports [24,42]. It seems that there was a discrepancy between the foliar starch content and A in the present study, because A was recorded as the highest of the three temperatures at elevated temperature I, whereas the starch content was not. This observation may be due to the higher growth rate and higher demand for energy and carbon skeletons of plants grown at elevated temperatures compared to those grown at ambient temperature. Thus, more starch was consumed by rapid plant growth at elevated temperatures, leaving fewer starch grains and soluble sugars to be stored in leaves [24,43]. This explanation could be supported by the interesting finding that there were more and larger mitochondria at elevated temperature I, because plants with higher growth rates have higher energy demands and more mitochondria--the organelles providing most of the ATP required for cell growth and maintenance through oxidative phosphorylation [42,44]. In addition, plants at elevated temperatures have a higher E in the darkness compared with those grown at ambient temperature; thus, more soluble sugars and starch will be consumed. Elevated temperatures profoundly affect plant respiration [1,45], but relatively little information exists on the underlying mechanism. Our current results suggest that elevated temperature regulates plant respiration probably by affecting mitochondrial number and size.

## Conclusions

In conclusion, we investigated the effects of experimental warming on leaf functional traits, leaf structure, and leaf biochemistry in *A. thaliana*, apart from fitness components. Several findings are worth noting. Firstly, moderate simulated climatic warming benefited *Arabidopsis *growth, whereas severe warming produced detrimental effects. This implies that global warming can have both beneficial and detrimental impacts on plants, especially on those belonging to the same functional group as *Arabidopsis*, i.e., moderate warming is beneficial to plants when it is below their optimum temperature, whereas higher levels of warming are detrimental to plants. Secondly, the increase in A we observed under moderately warm conditions may be attributed to the increase in SD, chlorophyll content, and number of chloroplasts. Thirdly, starch accumulation in chloroplasts may be the main factor influencing chloroplast ultrastructure, and elevated temperature regulates plant respiration by probably affecting mitochondrial size. Finally, high SOD and CAT activities may enable plants grown at elevated temperatures to exhibit relatively high tolerance to temperature stress, thus alleviating the harmful effects of superoxide anion radicals and hydrogen peroxide.

## Authors' contributions

BJ and NJT designed the experiments. LW, JW, KZJ, YW, XXJ, CYN, and YLW performed the experiments and analyzed the data. BJ and NJT analyzed the data and wrote the manuscript. All authors read and approved the final manuscript.
